# Assessing Tumor-Infiltrating Lymphocytes in Breast Cancer: A Proposal for Combining Immunohistochemistry and Gene Expression Analysis to Refine Scoring

**DOI:** 10.3389/fimmu.2022.794175

**Published:** 2022-02-11

**Authors:** Hanne Locy, Stefaan Verhulst, Wilfried Cools, Wim Waelput, Stefanie Brock, Louise Cras, Ann Schiettecatte, Jan Jonckheere, Leo A. van Grunsven, Marian Vanhoeij, Kris Thielemans, Karine Breckpot

**Affiliations:** ^1^ Laboratory for Molecular and Cellular Therapy, Department of Biomedical Sciences (BMWE), Vrije Universiteit Brussel (VUB), Brussels, Belgium; ^2^ Liver Cell Biology Research Group, BMWE, VUB, Brussels, Belgium; ^3^ Interfaculty Center Data processing and Statistics, VUB, Brussels, Belgium; ^4^ Department of Anatomo-Pathology, Universitair Ziekenhuis Brussel (UZ Brussel), Brussels, Belgium; ^5^ Department of Radiology, UZ Brussel, Brussels, Belgium; ^6^ Department of Surgery, UZ Brussel, Brussels, Belgium

**Keywords:** breast cancer, immunohistochemistry, gene expression profiling, tumor-infiltrating lymphocyte, immunotherapy targets

## Abstract

Scoring of tumor-infiltrating lymphocytes (TILs) in breast cancer specimens has gained increasing attention, as TILs have prognostic and predictive value in HER2^+^ and triple-negative breast cancer. We evaluated the intra- and interrater variability when scoring TILs by visual inspection of hematoxylin and eosin-stained tissue sections. We further addressed whether immunohistochemical staining of these sections for immune cell surface markers CD45, CD3, CD4, and CD8 and combination with nanoString nCounter^®^ gene expression analysis could refine TIL scoring. Formalin-fixed paraffin-embedded and fresh-frozen core needle biopsies of 12 female and treatment-naive breast cancer patients were included. Scoring of TILs was performed twice by three independent pathologists with a washout period of 3 days. Increasing intra- and interrater variability was observed with higher TIL numbers. The highest reproducibility was observed on tissue sections stained for CD3 and CD8. The latter TIL scores correlated well with the TIL scores obtained through nanoString nCounter^®^ gene expression analysis. Gene expression analysis also revealed 104 and 62 genes that are positively and negatively related to both TIL scores. In conclusion, integration of immunohistochemistry and gene expression analysis is a valuable strategy to refine TIL scoring in breast tumors.

**Graphical Abstract:**
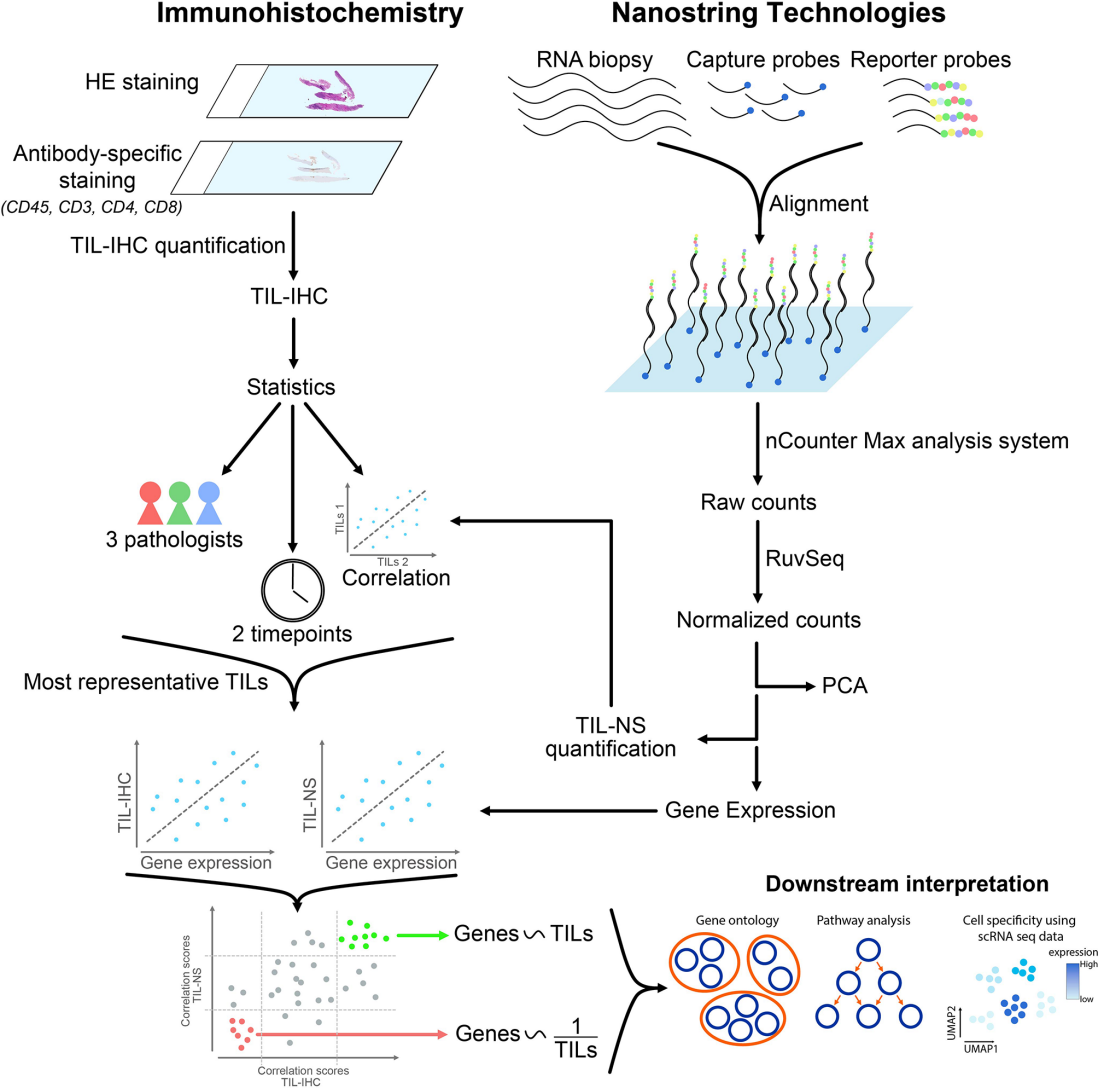


## Introduction

Globally, breast cancer is the most frequently diagnosed cancer and a major cause of death in women worldwide (1). Recurrence of breast cancer cells occurs in a significant number of patients with a recent meta-analysis showing that the overall 5-year rates for distant metastasis, regional recurrence, and local recurrence in <35-year-old breast cancer patients were 16.6%, 5.1%, and 6.7%, respectively (2). Currently, tumor-node-metastasis staging is implemented to stratify patients; yet, patients within the same stage still can show a different clinical outcome. This suggests that a complex and dynamic interaction occurs between tumor cells and the immune system at all stages (3). This hypothesis can be extended to the different clinical breast cancer subtypes, which are defined based on expression of hormone receptors and human epidermal growth factor receptor 2 (HER2) and on identification of transcriptional signatures (4), referring to luminal breast cancer [roughly equivalent to tumors expressing estrogen receptor (ER) and/or progesterone receptor (PR)], HER2^+^, and triple-negative breast cancer (TNBC), lacking expression of ER, PR, and HER2. It has been shown that different breast cancer subtypes are typified by a different immune contexture, considering the type of immune cells, their distribution, location, and presence in a tertiary lymphoid structure (TLS), density, and functional orientation (5). This reflects the heterogeneity of the disease, which is appreciated at various levels, from morphology to molecular alterations, with well-known genotypic-phenotypic correlations (6, 7).

The intra- and peritumoral breast cancer stroma contains immune cells and nonimmune cells, e.g., cancer-associated fibroblasts and adipocytes (7, 8). The importance of stromal biology in tumor progression is translated in a tumor stroma-based gene expression signature associated with clinical outcome (4, 9). This is not surprising as there are intricate interactions between cancer cells and tumor stroma, which overall promote tumor progression and are implicated in generating a therapy-resistant tumor environment (9, 10). There is culminating evidence that tumor-infiltrating lymphocytes (TILs) have a major effect on the clinical attributes of human cancer and can influence the tumor response to various therapy regimens. In particular, stromal TILs have been shown to have prognostic value in HER2^+^ breast cancer and TNBC. Increasing levels of TILs have been associated with improved therapy outcome in HER2^+^ breast cancer and/or TNBC patients treated with adjuvant (11–15), neoadjuvant chemotherapy (16–18) and monoclonal antibodies blocking programmed death-1 (PD-1) (19). The accumulating evidence that TILs are a potential biomarker in TNBC (and likely other breast cancer subtypes) further resulted in their designation as level 1B evidence and the proposal to include TIL reporting in clinical practice by the 16th St. Gallen International Breast Cancer Conference (20).

Scoring of TILs is mainly performed using the standardized method that was introduced by the International Immuno-Oncology Biomarker Working Group on Breast Cancer (tilsinbreastcancer.org). This immunohistochemistry method uses hematoxylin and eosin (HE)-stained tumor sections followed by visual inspection to score TILs (21). TILs are defined as mononuclear immune cells that infiltrate tumor tissue and constitute a continuous variable quantified as a percentage of area occupied by TILs per total stromal area. Including TILs as a biomarker in routine clinical practice requires that the scoring methodology shows little to no intra- and interrater variability. However, the heterogeneity of tumors makes visual scoring challenging. Moreover, scoring of TILs based on HE-stained specimens does not consider that mononuclear immune cells can have various origins and linked herewith various functions (22). Therefore, technologies that complement and/or refine TIL scoring merit exploration. In this regard, gene expression profiling (GEP) methods, such as nanoString nCounter^®^ panCancer Immune Profiling, developed to study the abundance and characteristics of tumor-infiltrating immune cells, are of interest.

We probed immunohistochemical analysis and nanoString nCounter^®^ panCancer Immune gene expression profiling as strategies to score TILs and gain insight into their heterogeneity. We posed the questions: What is the intra- and interrater variability between trained raters when scoring TILs on HE-stained tissue sections, the gold standard method? What is the concordance between the TIL score obtained *via* immunohistochemical analysis and nanoString nCounter^®^ gene expression profiling? What is the relationship between the TIL score and expression of specific genes?

Scoring of TILs was performed on breast cancer tissue sections obtained from 12 female and treatment-naïve patients, representing TNBC, ER/PR^+^, and HER2^+^ breast cancer patients. The intra- and interrater variability when TILs are scored using immunohistochemistry increased with increasing TIL numbers. This variability decreased when tissue sections where stained for CD3 or CD8. These immunohistochemistry-based TIL scores correlated well with the TIL scores obtained through nanoString nCounter^®^ gene expression profiling with gene expression analysis further revealing 104 and 62 genes that were directly and inversely correlated to the TIL scores, respectively. As such gene expression analysis provided additional information, integrating immunohistochemistry and gene expression profiling, therefore, provides a framework to refine TIL scoring.

## Materials and Methods

### Clinical Samples

Fresh 16 G × 100 mm or 18 G × 100 mm core needle biopsies (CNBs) from breast tumors were obtained from patients that were diagnosed at the University hospital of Brussel (UZ Brussel) from December 2017 to January 2020 and who gave informed consent. Fresh CNBs were collected in 50 ml tubes (62.547.254, Sarstedt, Nümbrecht, Germany) containing 5 ml RNA*later™* solution (R0901, Sigma-Aldrich, St. Louis, MO, USA). Samples were stored at 4°C for maximally 1 month before further processing. The project follows the Helsinki Declaration and was approved by the ethics council of the UZ Brussel (2017/344 and 2017/400).

### RNA Extraction From Core Needle Biopsies and Quality Control

Total RNA extraction from CNBs and quality controls (yield, integrity) were performed as described (23).

### Immunohistochemistry on Formalin-Fixed Paraffin-Embedded Tumor Samples

One to five CNBs were routinely obtained from patients for diagnostic purposes. In total, 37 biopsies were obtained for 12 patients. CNBs were processed to formalin-fixed paraffin-embedded (FFPE) specimens using the Sakura instrument (Tissue-Tek VIP^®^ 6AI Vacuum Infiltration Processor, Sakura, Brøndby, Denmark). Tumor biopsies were fixed using 10% formalin for 1.5 h at 35°C and dehydrated by immersing the tissue in different concentrations of ethanol for 4.5 h at 35°C. Next, xylene was used as a clearing agent for 2 h at 35°C. Finally, samples were paraffin embedded at 58°C for 3 h. FFPE-CNBs (3-µm-thick slides) were automatically stained with HE following the HE-staining protocol of Tissue-Tek^®^ Prisma. Additional slides were automatically stained (Benchmark Ultra instrument) with antibodies that specifically bind the surface markers: CD45 (9 µg/ml, 2B11PD7/26, Roche, Mannheim, Germany), CD3 (0.4 µg/ml, 2GV6, Roche), CD4 (2.5 µg/ml, SP35, Roche), or CD8 (0.35 µg/ml, SP57, Roche). Processing and staining of tumor samples were performed at the Anatomo-Pathology Department of the UZ Brussels.

### Molecular Classification of Breast Tumor Specimens

Molecular classification of FFPE specimens was performed according to the recommendations following the 13th St. Gallen International Breast Cancer Conference (2013) and was based on immunohistochemical measurement of ER, PR, ERBB2 (HER2), and Ki-67 with *in situ* hybridization confirmation when appropriate. Different subtypes are defined as follows: luminal A-like (ER^+^, PR^+^, HER2^−^, Ki67^low^, *n* = 5), HER2^+^ luminal B-like (ER^+^, HER2^+^, *n* = 1), HER2^−^ luminal B-like (ER^+^, HER2^−^, at least one of the following: Ki67^high^, PR^−^, or PR^low^, *n* = 3), HER2^+^ nonluminal (HER2^+^, ER^−^, PR^−^, *n* = 2), and TNBC (ER^−^, PR^−^, HER2^−^, *n* = 1).

### nanoString nCounter^®^ Gene Expression Profiling

RNA input (50 ng) for CNBs was calculated according to the nanoString input recommendations ([100/percent of sample >200 nt] × [recommended input amount] ng). Samples were hybridized according to manufacturer’s recommendations using the nCounter^®^ Human PanCancer Immune Profiling panel (NanoString Technologies, Seattle, WA, USA). Absolute counts were quantified by the nCounter digital analyzer (nCounter MAX Analysis System, located at BRIGHTcore facility at UZ Brussel). Quality control after analysis was performed using the nSolver analysis software 4.0. Raw counts were extracted from the software and further processed in R.

### Scoring of Tumor-Infiltrating Lymphocytes

Slides of FFPE-CNBs that were stained for immunohistochemical analysis were digitalized using the Panoramic SCAN I (3DHISTECH, Budapest, Hungary) instrument and uploaded in the Pathomation software (vub.pathomation.com). Test sessions were independently generated by a third party. Tissue section of 1–5 CNBs per patient were annotated separately. Biopsies were randomized by the software before scoring by three trained and blinded pathologists. TILs were scored independently by each pathologist based on visual inspection of tissue sections stained with HE [following recommendations published by Hendry et al. (21)] or stained with antibodies that specifically bind CD45, CD3, CD4, or CD8. The resulting scores are referred to as TIL-HE, TIL-CD45, TIL-CD3, TIL-CD4, or TIL-CD8, collectively called TIL-IHC. The three pathologists scored every biopsy twice with a washout period of 3 days. For all methods, TIL scores were reported for the stromal compartment (as percentage) within the tumor border. TILs present in tumor areas showing artifacts, necrosis, or hyalinization were excluded. In case of inspection of HE-stained sections, only mononuclear cells were considered, thus excluding polymorphonuclear cells. In case of inspection of antibody-stained sections, only cells detected by the antibody were considered. TIL scores based on gene expression profiling was calculated on normalized counts as described (24).

### Data Analysis and Statistics

The 37 CNBs were rated twice and independently by three trained pathologists (raters) to evaluate intra- and interrater variability (variances specific to time of measurement, method, and rater). Intrarater variability is visualized per method as the absolute differences between replicates in function to the averaged TIL score per patient. The interrater variability or variability of the TIL scores among three independent pathologists is represented as the standard deviation of average TIL scores, calculated for every CNB at two different time points (first or second score, referred to as set 1 (s1) and set 2 (s2)). The interrater variability is calculated for the TIL-scores obtained after visual inspection of sections stained with HE or stained with antibodies specific for CD45, CD3, CD4, or CD8. Interpretation of method variability per method is allowed by representing the range and quartiles of the averaged TIL scores over replications and raters in box plots. Dots reflect values outside the 1.5 interquartile range. Whiskers were drawn according to minimum-maximum method.

A mixed model on log-transformed ratings is used (R-package MethComp) for an estimation of the actual variances while allowing for a nonconstant bias (25, 26). Results include the rater-specific biopsy-method (IxM) and the biopsy-replication interactions (IxR) as well as the residuals. The IxR reflects intramethod variability. The IxM reflects intermethod variability. Treating the two replicates as different raters, the concordance correlation coefficient (CCC) is extracted from the variance components as 0.751, within the 95% confidence bounds (0.649–0.827).

nanoString nCounter^®^ gene expression analysis and visualization. Raw RCC files were imported in R-Studio and normalized using R-package RUVSeq (*k* = 1) (27). Differential expression between samples of high and low TIL-NS score was performed using DESeq2 (28). Principal component analysis was performed using base R-functions and visualized by R-package ggplot2. The TIL scores generated using the gene expression data were calculated as the sum of the average log2-normalized expression for all marker genes for each cell type and are referred to as TIL-NS. The final TIL score was calculated by averaging all the cell type scores whose correlations with CD45 (PTPRC) exceeded 0.6 (24). The following 10 cell types were included in the TIL scores: B cells (CD19, TNFRSF17), CD8^+^ T cells (CD8A, CD8N), cytotoxic cells (GNLY, GZMA, GMZB), DCs (CCL13, CD209), exhausted CD8^+^ cells (CD244, LAG3), macrophages (CD68), neutrophils (S100A12, CEACAM3), natural killer CD56^dim^ cells (IL21R), and T cells (CD3D, CD3E, CD3G). The Spearman correlation coefficient was calculated between nSolver and RUVSeq-derived TIL scores in R. Pearson correlation was determined between TIL-NS-RUVSeq and diverse TIL-IHC methods in R. All correlation analysis was visualized in scatter plots using R-package ggpubr. Creation of heatmaps was performed using R-package ComplexHeatmap and Venn diagrams using R-package eulerr.

### Data Availability

- Imaging dataset: Microscopical images of HE-, CD45-, CD3-, CD4-, and CD8-stained CNBs (http://minfx44.vub.ac.be:8081/omero)
- Raw and normalized nCounter Gene Expression Data: Gene expression of 12 breast cancer samples using nCounter Human PanCancer Immune Profiling panel, GEO database (GSE180370)
- RNA-Seq data [Azizi et al. (29)]: Gene expression of various immune cell populations, GEO-database (GSE114727)

## Results

### Staining of Tissue Sections With Immune Cell-Specific Antibodies Decreases the Inter- and Intrarater Variability of Immunohistochemistry-Based TIL scores

**Figure 1:**
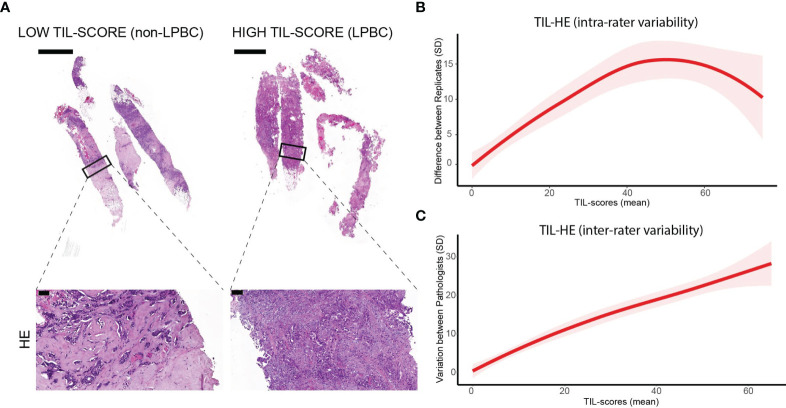
Intra- and interrater variability of TIL scores determined on HE-stained tissue sections. **(A)** HE staining of breast cancer tissue sections. Scale bars: 2 and 100 µm (respectively upper and lower panels). **(B)** Graph showing intrarater variability. **(C)** Graph showing interrater variability. Data information: 37 biopsies were stained with HE and scored twice with a washout period of 3 days by three independent pathologists. In **(B**, **C)**, data are presented as mean ± confidence interval of 95%.

Scoring of TILs was performed on 37 HE-stained tissue sections derived from tumors of 12 treatment-naive female patients. The histopathological and molecular subtypes as well as the Nottingham grade of the tumors are shown in **Supplementary Table S1**. TILs were defined within the tumor border as mononuclear cells and the TIL-HE, i.e., TIL score determined on HE-stained sections, was defined as the area occupied by TILs in the area of stromal compartment (percentage). Tissue sections representative for lymphocyte predominant breast cancer (LPBC) versus non-LPBC are shown in **Figure 1A**.

To study the interrater variability, TIL scoring was performed by three trained raters. To study intrarater variability, TILs were scored twice by each rater with a washout period of 3 days. If the same biopsy is not rated identically by the same rater using the same method, this suggests a lack of precision inherent to the rating. If the same biopsy is not rated identically by different raters and/or different methods, this suggests rater- and/or method-specific bias. The TIL score assigned by each rater to the 37 analyzed tissue sections is shown in **Supplementary Table S2**. **Supplementary Figures S1**, **S2** visualize all data and averages over replicates to interpret actual intra- and interrater variability, respectively. For the intrarater variability (**Supplementary Figure S1**), replicate ratings are paired, implying more consistent ratings when lines are more horizontal (allowing interpretation of intrarater agreement). Horizontal lines suggest consistent replicate ratings. Log-transformation of the data is performed, avoiding obscuration of the smaller differences in visualization and to focus on interrater variability (**Supplementary Figure S2**) more easily. **Figure 1B** shows that the variability in TIL score was less when one rater scored a specimen twice (intrarater variability), while **Figure 1C** shows that the TIL score assigned to each specimen vary considerably between raters (interrater variability).

**Figure 2:**
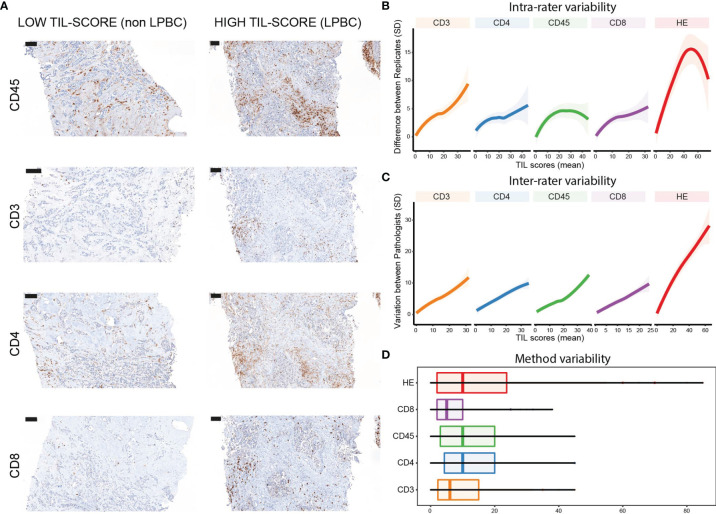
Intra- and interrater variability of TIL scores determined on antibody- and HE-stained tissue sections. **(A)** Staining of breast cancer tissue sections with antibodies specific for CD45, CD3, CD4, or CD8. Scale bars: 100 µm. **(B)** Graph showing intrarater variability. **(C)** Graph showing interrater variability. **(D)** Box plots showing method variability. Data information: 37 biopsies were stained with HE or immune cell-specific antibodies and scored twice with a washout period of 3 days by three independent pathologists. In **(B**, **C)**, data are presented as mean ± confidence interval of 95%.

Because of this variability, we explored if staining of tissue sections with antibodies that specifically bind immune cell surface markers, particularly CD45, CD3, CD4, and CD8, would allow more reproducible TIL scoring. **Figure 2A** shows staining of tissue sections classified as non-LPBC versus LPBC with these antibodies. Different methods that are used to rate TIL levels should ideally result in similar ratings, and variability in ratings between and within raters should be considered. To evaluate the different methods, we focused on agreement of the assigned TIL score. Matching intra- and interrater agreement would imply horizontal lines at zero. We observed that intrarater (**Figure 2B** and **Supplementary Figure S1**) and interrater (**Figure 2C** and **Supplementary Figure S2**) variability was lower for tissue sections that have a low TIL score compared with tissue sections that have a high TIL score when using tissue sections stained with immune cell-specific antibodies. In contrast, TIL scores assigned using the standard method (TIL-HE) shows larger differences between raters and replicates irrespective of the TIL-level estimate, suggesting that TIL-HE is less favorable for rating TIL scores. **Figure 2D** and **Supplementary Figure S3** visualize the method-specific variability of averaged and individual TIL scores, respectively. Results of a mixed model on log-transformed ratings to estimate intra- and intermethod variabilities are represented in **Supplementary Table S3**.

### TIL Scores Determined Using Immunohistochemically Stained Tissue Sections and nanoString nCounter^®^ Gene Expression Profiling Correlate Well

**Figure 3:**
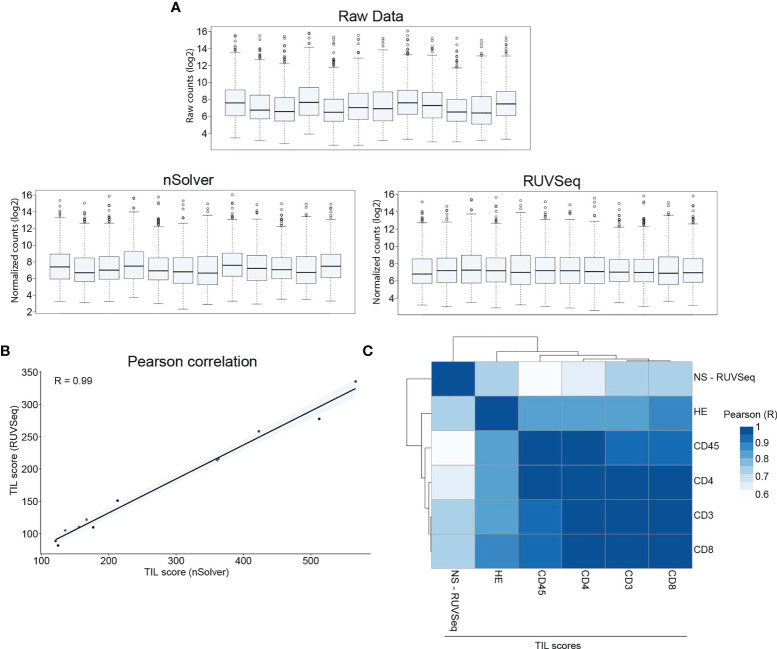
TIL score calculated on RUVSeq-normalized expression data correlates with TIL-score based on immunohistochemistry. **(A)** Box plots showing raw counts obtained from nCounter MAX analysis, normalized counts using nSolver software, and normalized counts using RUVSeq package. **(B)** Graph showing the correlation between nSolver- or RUVSeq-calculated TIL scores. **(C)** Heatmap showing Pearson correlation between different TIL scoring methods. Data information: 12 breast cancer CNB samples were used to extract RNA for downstream gene expression profiling.

Total RNA was extracted from CNBs. The RNA quality measures, including size distribution, RNA-integrity number (RIN), and DV200 values (percentage of RNA fragments with a length >200 nucleotides) are shown in **Supplementary Table S4**. This RNA was subjected to nanoString nCounter^®^ panCancer Immune gene expression profiling. Normalization of raw RNA counts using nSolver software was not satisfactory (**Figure 3A**). Therefore, we used an alternative approach by normalizing raw counts using the RUVSeq package (30). TIL scores were calculated using normalized counts from the nSolver and RUVSeq analysis and the cell type profiling algorithm described by Danaher et al. (24). We observed a strong correlation between both TIL scores (Pearson *R* = 0.99, **Figure 3B**), suggesting that using different normalization algorithms does not impact the TIL-score calculations. Still, downstream analysis needs to be performed on efficiently normalized data. We addressed whether TIL scores assigned by immunohistochemical analysis (TIL-IHC), comparing staining with HE or immune cell-specific antibodies, correlated with the TIL score assigned nanoString gene expression profiling (TIL-NS-RUVSeq). We calculated the Pearson correlation coefficients and observed that the TIL score assigned during immunohistochemical analysis based on the HE, anti-CD3, and anti-CD8 antibody staining correlated the highest (>0.7) with the TIL score assigned based on the nanoString gene expression profiling (TIL-NS) (**Figure 3C**).

### Integrating Immunohistochemistry and Gene Expression Profiling Provides a Broad View on the Breast Tumor Immune Environment

**Figure 4:**
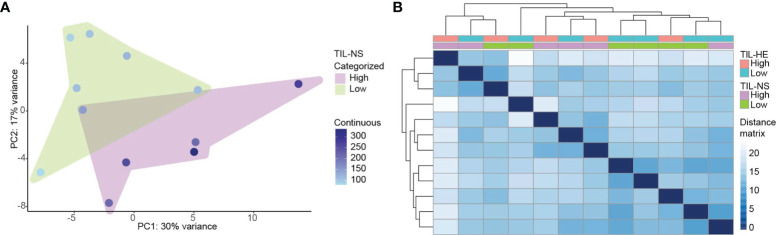
Specimens with a low and high TIL-NS show overlapping gene expression. **(A)** Principal component analysis of samples clustered on low and high TIL-NS. The color of the dots represents the TIL-NS score. **(B)** Distance matrix of samples with hierarchical clustering. Color bars represent low or high TIL-HE or TIL-NS scores.

We addressed the relationship between the TIL score and gene expression profiling using the RUVSeq-normalized gene expression data. We performed multidimensional reduction analysis using principal component analysis to visualize the variation between different breast cancer specimens, showing that breast cancer specimens with low and high TIL-NS (with a cutoff value of 250 normalized counts) were not completely separated (**Figure 4A**). Hierarchical clustering based on the expression of all genes within each patient corroborated these findings, supporting that breast cancer specimens cannot be classified into two groups (TIL-HE cutoff value of 15) and that TILs needs to be considered a continuous variable (**Figure 4B** and **Supplementary Figure S4**).

**Figure 5:**
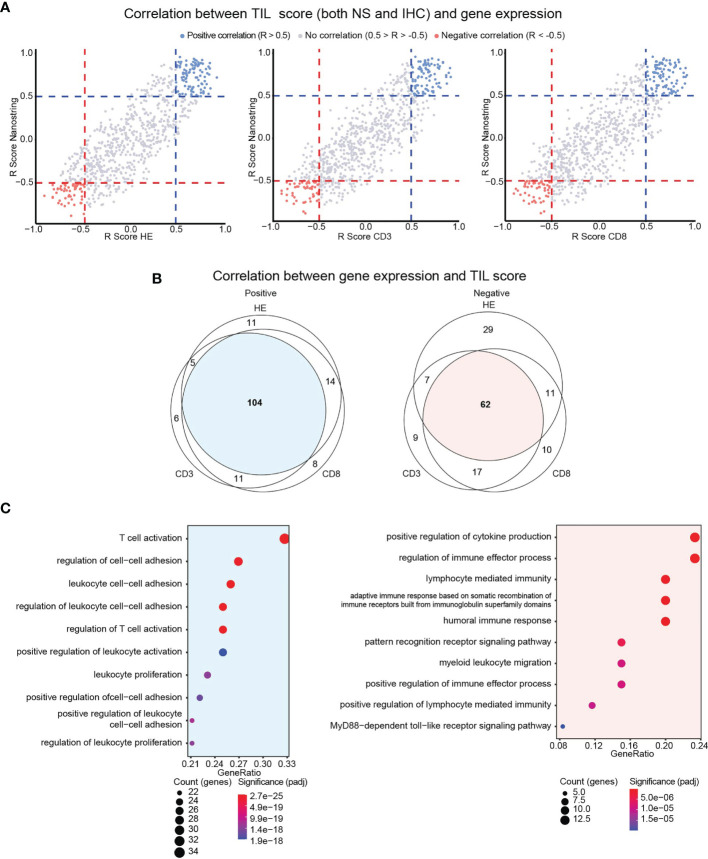
Identification of genes directly or inversely correlated with TIL scores. **(A)** Scatter plot showing correlation (*R* scores) between gene expression and TIL scores for TIL-HE, TIL-CD3, and TIL-CD8 methods. **(B)** Venn diagram showing overlap in directly or inversely correlated genes between different TIL-scoring methods. **(C)** Gene ontology analysis showing functions in which communal genes are involved. Data information: Positive correlation defined as *R* > 0.5, negative correlation defined as *R* < −0.5.

We therefore scrutinized if genes are directly or inversely correlated with the TIL score. We performed Spearman correlation analysis between the expression of single genes versus TIL-IHC or TIL-NS. The correlation coefficients were plotted in a scatter plot that was split in different sections (**Supplementary Figure S5**). As shown in **Figure 5A**, we identified genes that were directly (in red, *R* > 0.5) or inversely (in blue, *R* < −0.5) correlated with TIL scores based on TIL-NS versus TIL-CD3, TIL-CD4, and TIL-CD8. We did not detect genes that were directly correlated with a TIL-score of one method and inversely with a TIL-score of another method, confirming accordance between TIL-scoring methods. Of the genes that were directly and inversely correlated with the scores of TIL-NS versus TIL-CD3, TIL-CD4, or TIL-CD8 respectively, 104 and 62 genes were shared (**Figure 5B** and **Supplementary Table S5**). Genes that directly correlated to a TIL score are linked to T-cell/leukocyte activation, proliferation, and cell adhesion, while genes that inversely correlated with a TIL score are linked to innate signaling, myeloid cell migration, and mostly humoral responses (**Figure 5C**). A list of the top 20 of biological activities with involved genes is shown in **Supplementary Tables S6, S7**.

### Gene Expression Analysis Provides Insight Into the Cell Types Associated With a High TIL Score

**Figure 6:**
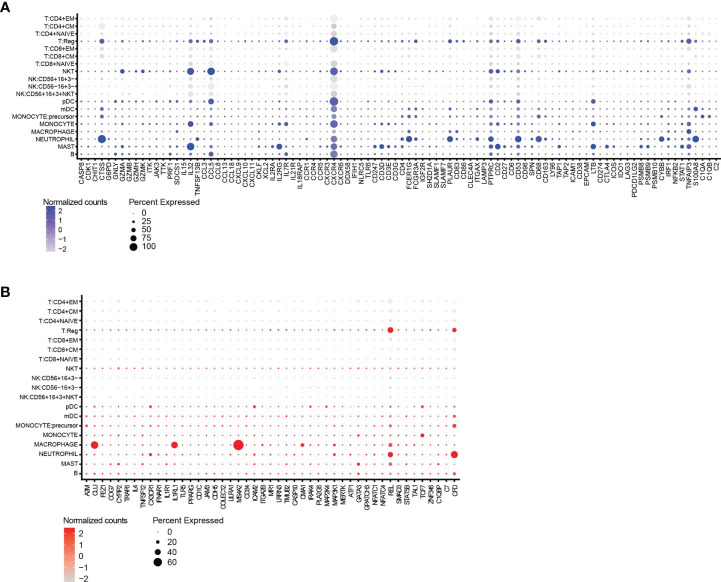
Gene expression provides insight into the cell types associated with a high TIL-score. **(A)** The split dot blot shows immune cell types (*y*-axis) and genes (*x*-axis) that are directly correlated with the TIL scores. Genes that are not expressed in any cell population are not plotted. **(B)** The split dot blot shows immune cell types (*y*-axis) and genes (*x*-axis) that are inversely correlated with the TIL scores. Genes that are not expressed in any cell population are not plotted. Data information: the size of the circle represents the percentage of cells showing expression, while the color code represents the normalized counts of every gene.

We scrutinized a publicly available scRNAseq dataset with a high number of immune cell populations from breast tumors to evaluate if the genes identified as directly or inversely correlated can be linked to specific immune cell types (GEO database, GSE114727) (29). **Figure 6** depicts genes, which are either directly (**Figure 6A**) or inversely (**Figure 6B**) correlated with TIL scores, were associated with various immune cell subsets, but mainly regulatory T cells (Tregs) and myeloid immune cells such as mast cells, macrophages and neutrophils were identified as cells that score high for several of the genes that are positive correlated to a TIL score.

## Discussion

In this study, the reproducibility of TIL scoring using the standard method described by Hendry et al. (21) was compared with TIL scoring on tissue sections stained with immune cell-specific markers: CD45, CD3, CD4, and CD8 and TIL scoring based on nanoString nCounter^®^ panCancer Immune gene expression profiling. Variability in TIL scoring was observed among pathologists as well as among different time points of scoring by one pathologist when HE-stained tissue sections were used with variability observed across all sections. This variability is in contrast to earlier reports that show inter- and intrarater agreement between pathologists when assessing TILs according to the standardized TIL-scoring method (31–33). The latter might be a result of the larger number of raters or the evaluation of TILs within one breast cancer subtype in these studies. In practice, TIL scoring is becoming increasingly relevant for different cancer types and TIL scoring is performed by a limited number of pathologist, so we argue that our study using a heterogenous sample set and three raters is more closely resembling the expected outcome of routine daily practice. Hence, the observed variability in TIL-HE justified research in methods to refine the methodology of TIL scoring.

Consistency in TIL scoring improved when tissue sections were stained with antibodies for specific immune cell types, e.g., CD8^+^ T cells. Regardless, the variability of TIL scores given to sections that have higher TIL levels was still considerable. This led us to the conclusion that TIL scoring based on immunohistochemical staining of tissue sections followed by visual inspection, although benefitting from immune cell type staining, is best combined with an additional TIL-scoring method. This method should circumvent the pitfalls of immunohistochemistry-based TIL scoring, among which introduction of bias because of manual preselection of representative tumor regions for each slide. To that end, image processing and machine learning are being implemented and have been shown to improve TIL scoring (34, 35). Yet, TIL scoring is still performed on selected slides, which might not be representative for the entire biopsy and heterogeneity of the tumor. Also, the number of stainings for specific immune cells that can be performed on FFPE samples is restricted by the amount of material, as multiplex immunohistochemistry is not routinely performed. Therefore, we selected nanoString nCounter^®^ panCancer Immune gene expression profiling as a method that could potentially refine and complement TIL-IHC. Indeed, this method can be performed with RNA extracted from FFPE tissue used for TIL-IHC, can be automated and allows TIL scoring based on a diverse yet still comprehendible set of 770 genes. Moreover, the complementarity of nanoString nCounter^®^ gene expression analysis with immunohistochemistry has been shown while investigating immune gene profiles associated with breast cancer cohorts characterized by absence versus presence of nonactive or active TLS (36). Here, we observed that TIL-HE, TIL-CD3, and TIL-CD8 showed high correlation coefficients with TIL-NS, suggesting that variability in TIL-IHC could be resolved when complemented with TIL-NS.

We attempted to classify patients into a category with a low versus high TIL score. However, we observed in literature that the definition of a low versus a high TIL score varies considerably between research groups and institutions. For instance, Kurozimi et al. (37) define TILs categorically with low, intermediate, and high TILs defined as <10%, ≥10% and ≤40%, and >40%, respectively. Denkert et al. (18) defines low as 0%–10%, intermediate as 11%–59%, high TILs as ≥60%, while Loi et al. (38) and Hendry et al. (21) use a classification into non-LPBC or LPBC, the latter defined as ≥50% or variably defined as >50% or >60%. To conclude, there is no consensus on the cutoff for a high TIL-score with current studies ranging between >40% and ≥60%. We decided to approach infiltration of TILs as a continuous variable, in analogy to Kos et al. (39), as we observed in principal component analysis that classifying patients into patients with a low versus a high TIL score was not justified. Also, TIL-IHC did not allow classification into categories. We applied reversed reasoning and looked into genes that are either positively correlated or negatively correlated with both TIL-IHC and TIL-NS, meaning that when TIL scores are high/low with both TIL-scoring methods genes are also highly/lowly expressed or when TIL scores are high/low, genes are lowly/highly expressed, respectively. Since TILs have been shown to have prognostic as well as predictive value in mainly HER2^+^ breast cancer and TNBC patients, these genes might have similar value, although validation assays need to be performed to substantiate their significance. Analysis of the 104 genes that were positively correlated with the TIL score obtained *via* TIL-IHC and TIL-NS showed that these genes are differentially expressed between the immune hot, intermediate, and cold clusters defined by Tekpli et al. (40). These genes were higher expressed in hot tumors when compared with tumors classified in intermediate poor prognosis immune cluster. Conversely, analysis of the 62 genes that were inversely correlated with the TIL score showed that the majority (60% of the differentially expressed genes) were lower expressed in hot tumors when compared with tumors classified in the cold or intermediate poor prognosis immune cluster, warranting further analysis of these genes and their potential significance. Therefore, we used an existing scRNAseq dataset to identify which immune cell populations express the highest levels of these genes. This analysis does not probe TIL abundance rather provides insight in potential TIL composition and function. We observed that genes were mainly expressed in myeloid cells, such as neutrophils and macrophages and in Tregs. These cells are often linked to tumor progression (10, 41, 42). However, neutrophils and macrophages can take on different roles in the tumor microenvironment. Indeed, when classically activated, these cells can help kill cancer cells (43, 44). This dual role is a testimony to the heterogeneity and plasticity of these cells. We observed that highly expressed genes in myeloid cells in tumors with a high TIL score are indicative of their polarization towards a classically activated phenotype, e.g., CTSS, FcER1G, PLAUR, CYBB, and PTPRC (45–49), while the genes that were highly expressed in myeloid cells in tumors with low TIL scores were indicative of the tumor-promoting activity of these cells, as exemplified by CFD, CLU, IL1R, and MS4A2 (50–59). This observation argues that gene expression profiling and analysis can provide information on the functionality of cells and is therefore justified as a strategy to refine and complement TIL scoring using immunohistochemistry.

## Data Availability Statement

The datasets presented in this study can be found in online repositories. The names of the repository/repositories and accession number(s) can be found below: Raw and normalized nCounter Gene Expression Data: Gene expression of 12 BC-samples using nCounter Human PanCancer Immune Profiling panel, GEO database (GSE180370); RNA-Seq data [Azizi et al. (29)]: Gene expression of various immune cell populations, GEO database (GSE114727). Request for imaging dataset: Microscopical images of HE, CD45, CD3, CD4, CD8 stained CNBs can be directed to the corresponding authors.

## Ethics Statement

The studies involving human participants were reviewed and approved by the Ethics Council of the UZ Brussel (2017/344 and 2017/400). The patients/participants provided their written informed consent to participate in this study.

## Author Contributions

KT and KB conceptualized experiments, acquired funding, and managed and supervised the project. HL, SV, and WC performed data curation and data analysis, developed and/or designed methodology, and visualized data. HL and SV managed the project and validated obtained data. WW, SB, and LC performed TIL scoring and collected raw data. AS, JJ, and MV provided patient material. HL, SV, and KB wrote the manuscript. KT and LG reviewed and edited the manuscript. All authors proofread the manuscript. All authors listed have made a substantial, direct, and intellectual contribution to the work and approved it for publication.

## Funding

HL is supported financially by the “Vlaams Agentschap Innoveren & Ondernemen” (VLAIO grant number HBC 2017.0564). SV is a junior postdoctoral fellow of the Research Foundation Flanders (FWO-V grant number 1243121N). Research at the Laboratory for Molecular and Cellular Therapy is further supported by “Kom op tegen Kanker (Stand up to Cancer): the Flemish Cancer Society”, “Stichting tegen Kanker”, FWO-V, Innoviris, and the Research Council of the VUB (OZR) *via* a strategic research program (SRP48).

## Conflict of Interest

The authors declare that the research was conducted in the absence of any commercial or financial relationships that could be construed as a potential conflict of interest.

## Publisher’s Note

All claims expressed in this article are solely those of the authors and do not necessarily represent those of their affiliated organizations, or those of the publisher, the editors and the reviewers. Any product that may be evaluated in this article, or claim that may be made by its manufacturer, is not guaranteed or endorsed by the publisher.
