# Supplementary material for: Assessing Tumor-Infiltrating Lymphocytes in Breast Cancer: A Proposal for Combining Immunohistochemistry and Gene Expression Analysis to Refine Scoring
Source: Front Immunol. 2022 Feb 11;13:794175. doi: 10.3389/fimmu.2022.794175 (PMC8876933; doi:10.3389/fimmu.2022.794175)
Supplement: Supplementary file 1 [file DataSheet_1.docx]

Supplementary Material

**Supplementary Table S1: Overview of the clinical characteristics of the 12 female treatment naive patients from whom core needle biopsies were used throughout this study.**

**Table S1: Overview of the clinical characteristics of the 12 female treatment naive patients from whom core needle biopsies were used throughout this study.** The histopathological and molecular subtype as well as Nottingham grade are shown.

**Supplementary Table S2: Overview of TIL-scores based on visual inspection of H&E-stained tissue sections.**

**Table S2: Overview of TIL-IHC scores based upon immunohistochemical staining**. Patients are listed as specimen number, tissue section number refers to the respective core needle biopsy, raters refer to 3 independent trained pathologists who scored every biopsy twice (measurement 1 and 2) for 5 TIL-IHC methods (TIL-HE, TIL-CD45, TIL-CD3, TIL-CD4, TIL-CD8) in total.

**Supplementary Table S3: Estimation of variabilities: variances and conversion rules to TIL-HE per rater.**

**Supplementary Table S3: Estimation of variabilities.** Variance components estimates for the mixed model of log transformed ratings (left). IxR reflects intra-method variability. IxM reflects inter-method variability. Res stands for residuals. Conversion rules from each of the TIL-IHC methods towards the TIL-HE (TIL-HE ~alpha + beta *TIL-…).

**Supplementary Table S4: Quality control of the extracted total RNA.**

**Supplementary Table S4: Quality parameters of the extracted total RNA.** Per patient, concentration in (ng/µL), absorbance at 260 and 280nm, RNA integrity number (RIN) value and the DV200 values (percentage of RNA fragments with a length > 200 nucleotides) is determined to establish input levels for downstream GEP.

**Supplementary Table S5: List of genes that correlate with the TIL-IHC and TIL-NS score.**

**Table S5:** The table lists 104 and 62 genes that positively or negatively correlate with the TIL-IHC and TIL-NS score.

**Supplementary table S6: Genes positively correlated with the TIL-score – top 20 functions.**

**Supplementary table S6: Top 20 functions of genes positively correlated with TIL-score.** Table including GO-number with related function, gene ratio indicating the percentage of total differential expressed genes in the given GO-term, Q-value estimating false discovery rate, identification and number of involved genes.

**Supplementary table S7: Genes negatively correlated with TIL**-**score – top 20 functions.**

**Supplementary table S7: Top 20 functions of genes negatively correlated with TIL**-**score.** Table including GO-number with related function, gene ratio indicating the percentage of total differential expressed genes in the given GO-term, Q-value estimating false discovery rate, identification and number of involved genes.

**Supplementary Figure S1: Intra-rater variability.**

**
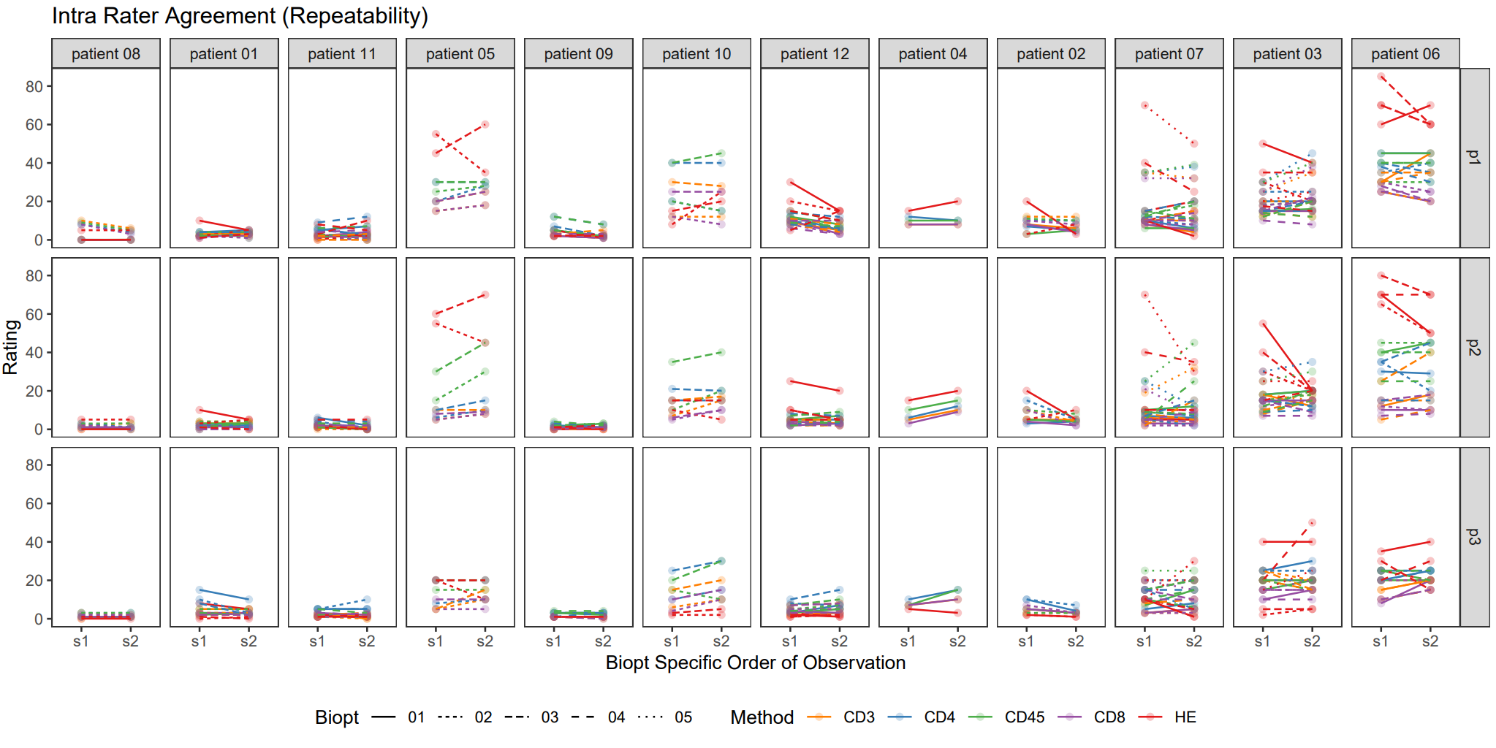
**

**Supplementary Figure S1: Graph summarizing intra-rater variability or repeatability.** Trellis plot combining changes in ratings (interaction plot) for a particular patient within a panel. Patients are indicated on top of the graph. Their order is determined by their increasing nanoString-based TIL-score from left to right. The three raters are indicated as p1, p2, p3 and represented in a horizontal way. Biopsies are scored by every rater twice (set 1 (s1) and 2 (s2) on x-axis) and are each individually represented with different line styles. Different TIL-scoring methods are indicated by different colors. Since the same biopsy should result in the same rating, when performed by the same rater or method, lines between s1 and s2 should be ideally horizontal.

**Supplementary Figure S2: Inter-rater variability.**


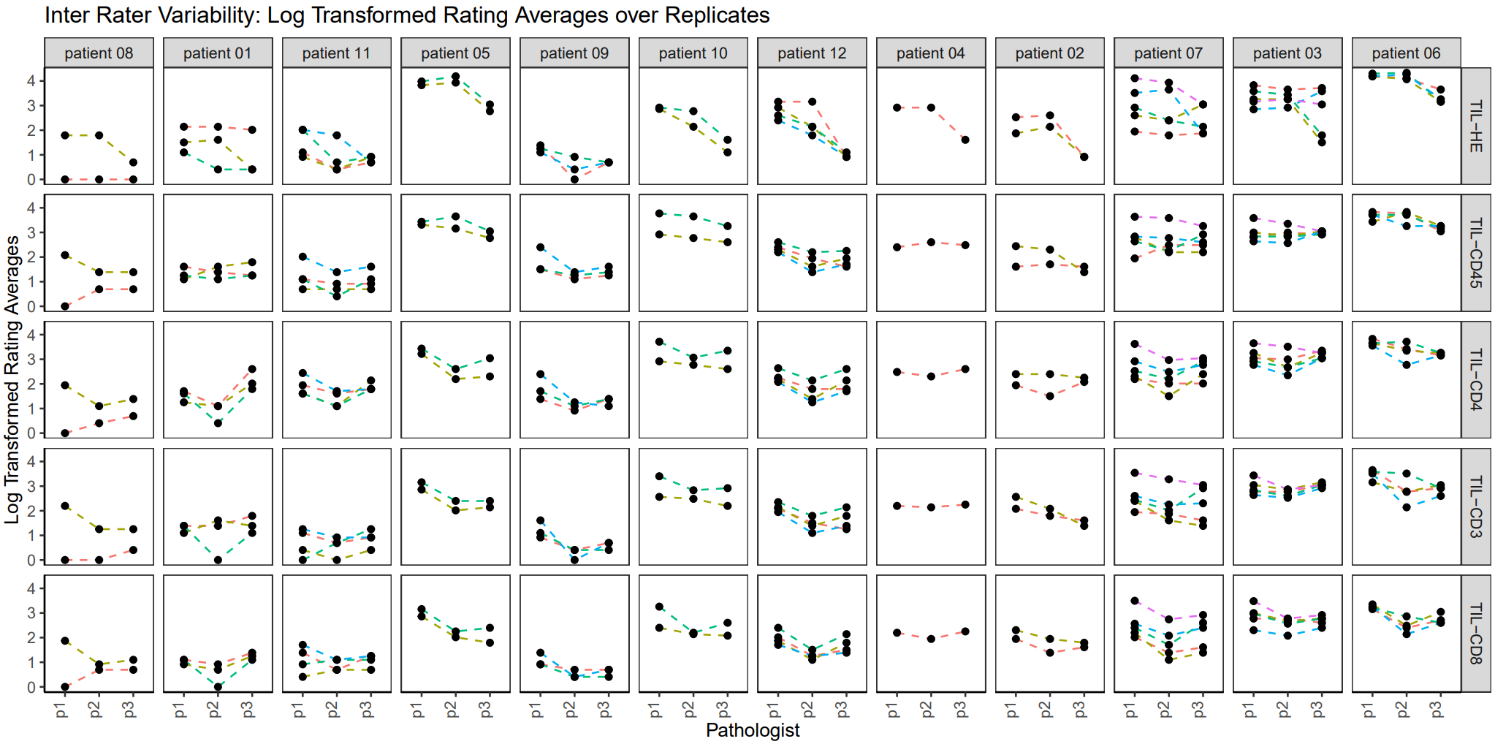


**Supplementary Figure S2: Inter-observer variability.** This graph represents the average log-transformed TIL-scores per biopsy over replicates (dots) of the same biopsy linked (lines) over raters (x-axis) within each panel. Panels are specific for a TIL-IHC method (row) and a patient (column). The order of patients is determined by their increasing nanoString-based TIL-score from left to right.

**Supplementary Figure S3: Method variability.**


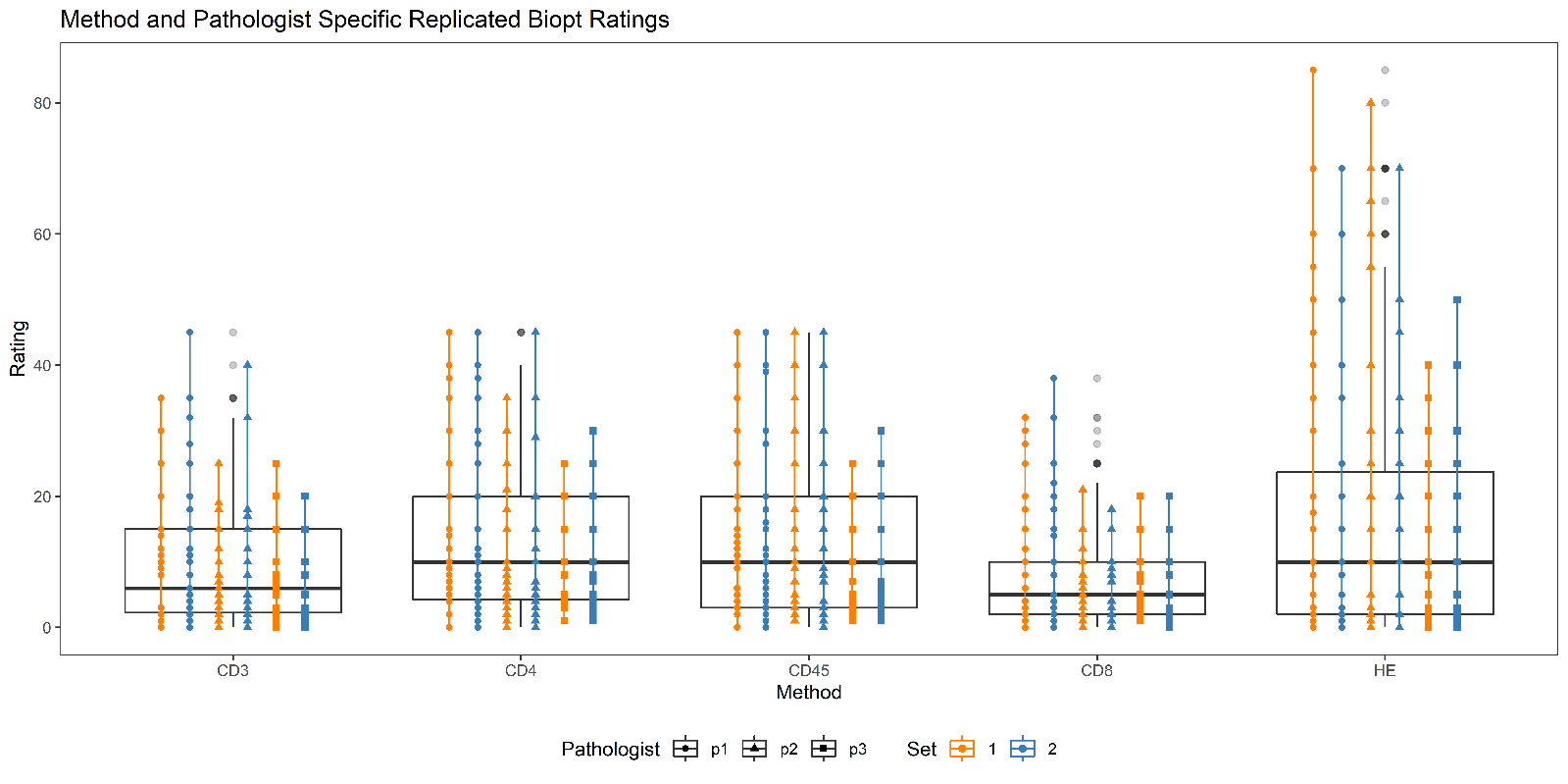


**Supplementary Figure S3: Graph summarizing method variability.** This graph represents the ratings and their summarized distribution (box plot) for the different methods (depicted on x-axis). Every datapoint represents the TIL-score of a particular used scoring method of 1 biopsy, resulting in n=37 (sometimes overlapping) datapoints on the vertical lines. Each vertical line is specific to a rater – replicate combination (raters indicated as p1, p2, p3 by respectively a circle, triangle and square symbol and replicates, s1 and s2, represented in orange and blue).

**Supplementary Figure S4: Differential expression between LPBC and non-LPBC.**


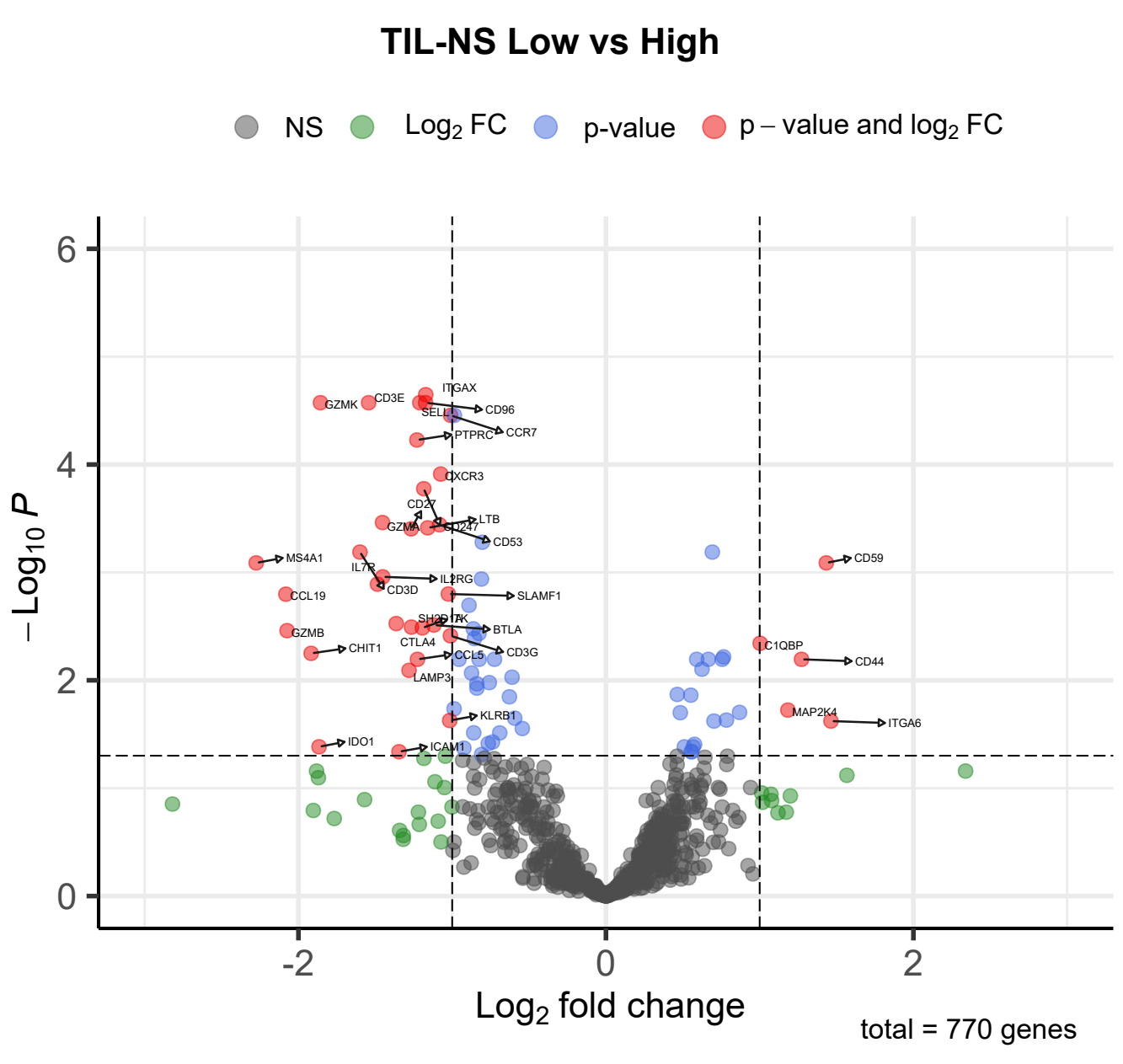


**Supplementary Figure S4: Volcano plot summarizing differentially expressed genes between TIL-NS low and high score.** Cut-off between low and high TIL-score was determined at 250 normalized counts. Genes that are statistically significantly different in expression (p-value <0.05) are depicted in red.

**Supplementary Figure S5: Validation of TIL-IHC and TIL-NS correlation scatter plot.**

**
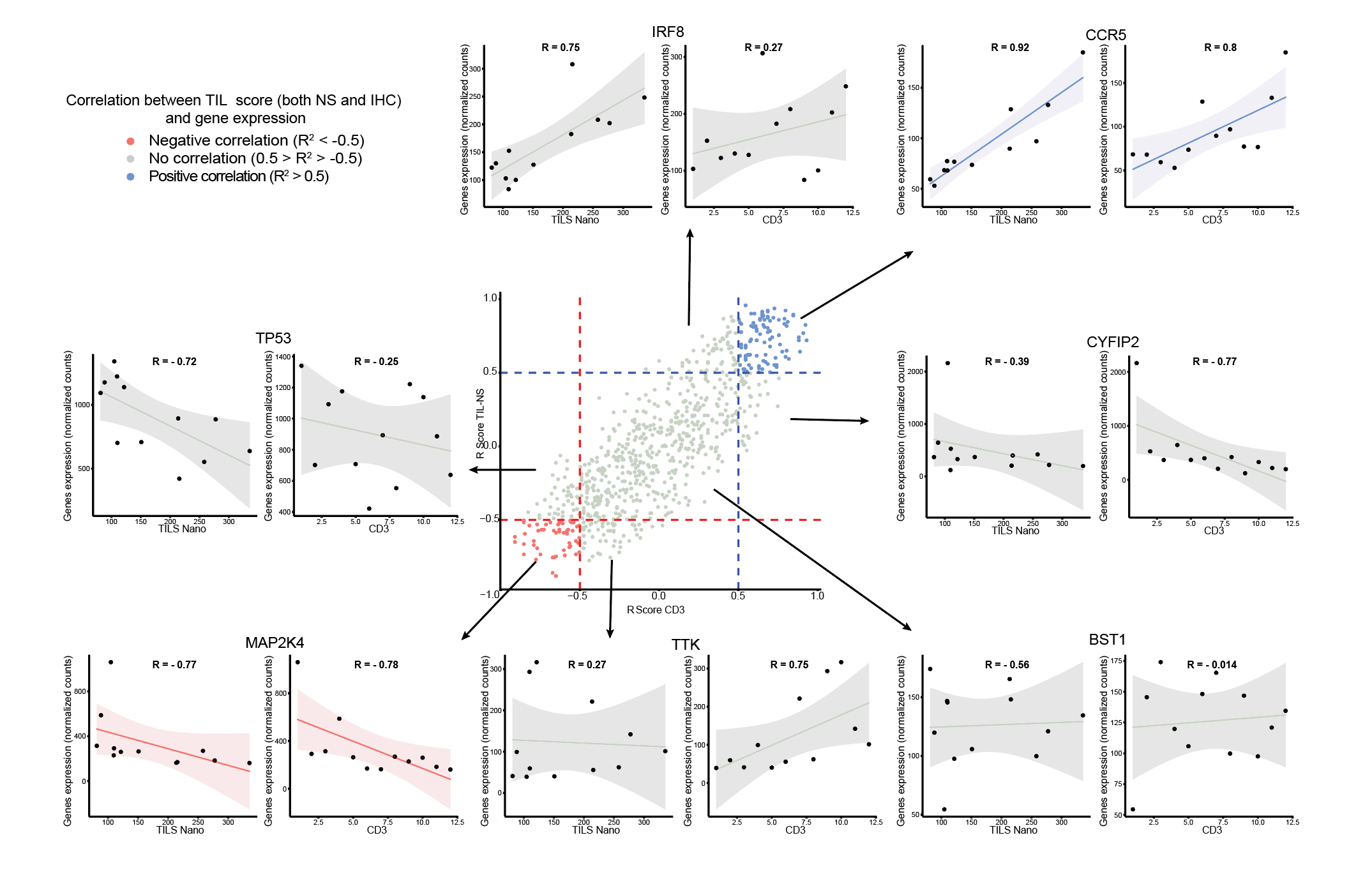
**

**Supplementary Figure S5: Correlation scatter plot between TIL-score and gene expression.** Summary of all the Spearman correlation coefficients is presented in a scatter plot which could be divided in different sections. Random genes per section were selected and correlation coefficient was depicted for as well as TIL-NS as various TIL-IHC scores. Here, the TIL-CD3 method is represented. None of the genes showed inverse correlations with the TIL-IHC method and TIL-NS, the IRF8 gene showed a positive correlation with the TIL-NS score, the CCR5 gene showed a positive correlation with both the TIL-IHC and TIL-NS method (indicated in blue), the TP53 gene showed a negative correlation with the TIL-IHC score, the BST1 gene (present at the bulk of genes) showed no correlation with both TIL-scoring methods, the CYFIP2 gene showed positive correlation with the TIL-IHC, the MAP2K4 gene belonged to genes that show negative correlation with both TIL-scoring methods (indicated in red), the TTK gene showed negative correlation with the TIL-NS score and finally, none of the genes showed positive correlation with the TIL-NS method and negative correlation with the TIL-IHC method. Negative correlation is defined as R²<-0.5, lack of correlation as 0.5>R²>-0.5, positive correlation as R²>0.5.
